# Detection of genomic alterations in breast cancer with circulating tumour DNA sequencing

**DOI:** 10.1038/s41598-020-72818-6

**Published:** 2020-10-08

**Authors:** Dimitrios Kleftogiannis, Danliang Ho, Jun Xian Liew, Polly S. Y. Poon, Anna Gan, Raymond Chee-Hui Ng, Benita Kiat-Tee Tan, Kiang Hiong Tay, Swee H. Lim, Gek San Tan, Chih Chuan Shih, Tony Kiat-Hon Lim, Ann Siew-Gek Lee, Iain Beehuat Tan, Yoon-Sim Yap, Sarah B. Ng

**Affiliations:** 1 Genome Institute of Singapore (GIS), Agency for Science, Technology and Research (A*STAR), Singapore, 138672 Singapore; 2 Division of Medical Oncology, National Cancer Centre Singapore (NCCS), Singapore, 169610 Singapore; 3 Department of General Surgery, Singapore General Hospital (SGH), Singapore, 169608 Singapore; 4 Vascular and Interventional Radiology Department, Singapore General Hospital (SGH), Singapore, 169608 Singapore; 5 KK Breast Centre, Kandang Kerbau Women’s and Children’s Hospital, Singapore, 229899 Singapore; 6 Department of Anatomical Pathology and Translational Pathology Centre, Singapore General Hospital (SGH), Singapore, 169608 Singapore; 7 Division of Cellular and Molecular Research, Humphrey Oei Institute of Cancer Research, National Cancer Centre Singapore (NCCS), Singapore, 169610 Singapore; 8 Department of Physiology, Yong Loo Lin School of Medicine, National University of Singapore (NUS), Singapore, 117597 Singapore; 9 SingHealth Duke-NUS Oncology Academic Clinical Programme (ONCO ACP), Duke-NUS Medical School, Singapore, 169857 Singapore; 10 Division of Surgical Oncology, National Cancer Centre Singapore (NCCS), Singapore, 169610 Singapore; 11 Department of General Surgery, Sengkang General Hospital, Singapore, 544886 Singapore

**Keywords:** Next-generation sequencing, Breast cancer, Cancer genomics, Genome informatics

## Abstract

Analysis of circulating cell-free DNA (cfDNA) has opened new opportunities for characterizing tumour mutational landscapes with many applications in genomic-driven oncology. We developed a customized targeted cfDNA sequencing approach for breast cancer (BC) using unique molecular identifiers (UMIs) for error correction. Our assay, spanning a 284.5 kb target region, is combined with a novel freely-licensed bioinformatics pipeline that provides detection of low-frequency variants, and reliable identification of copy number variations (CNVs) directly from plasma DNA. We first evaluated our pipeline on reference samples. Then in a cohort of 35 BC patients our approach detected actionable driver and clonal variants at low variant frequency levels in cfDNA that were concordant (77%) with sequencing of primary and/or metastatic solid tumour sites. We also detected *ERRB2* gene CNVs used for HER2 subtype classification with 80% precision compared to immunohistochemistry. Further, we evaluated fragmentation profiles of cfDNA in BC and observed distinct differences compared to data from healthy individuals. Our results show that the developed assay addresses the majority of tumour associated aberrations directly from plasma DNA, and thus may be used to elucidate genomic alterations in liquid biopsy studies.

## Introduction

One of the key objectives in precision oncology is to deliver better cancer diagnosis and tailored treatment. So far, analysis of tissue biopsy data is widely used to characterize tumour genomic landscapes, and to identify actionable somatic alterations^1,2^. However, tissue biopsies are invasive with constraints on frequency of tissue sampling, and may not be representative of the entire tumour load^3^.

As an alternative, recent studies have demonstrated the translational potential of circulating cell-free DNA (cfDNA), or circulating tumour DNA (ctDNA) in cancer patients, for improving cancer management^3,4^. Such liquid biopsy data measured directly from body fluids (e.g. plasma) can be used to detect tumour somatic alterations, with the ability to provide early prognostication and/or better molecular profiling of patients with cancer without the risk and discomfort of invasive biopsies^5^. For example, estrogen receptor 1 (*ESR1*) mutation detected in the ctDNA of breast cancer (BC) patients pre-treated with aromatase inhibitors correlated with inferior treatment outcome on exemestane, but not on fulvestrant^6,7^. *PIK3CA* mutation status based on ctDNA has also been demonstrated to predict benefit from *PI3K* inhibitor therapy in BC^8,9^. In other cancer types such as lung, ctDNA testing for *EGFR* mutation status has been approved by the Food and Drug Administration (FDA) to guide selection of therapy.

Several genetic techniques including digital droplet PCR (ddPCR) and BEAMing have been extensively applied to detect single nucleotide variations (SNVs) in cfDNA with very high precision (e.g. detection of alleles at lower than 0.1% frequency), but the analysis is restricted only to a limited number of genomic loci^5^. More recently, improvements in next generation sequencing (NGS) have allowed screening of broader genomic regions and simultaneous monitoring of multiple tumour-specific alterations in a single assay^2,10–12^. However, analyses of tumour NGS data from cfDNA is challenging due to several biological reasons (e.g. low cfDNA abundance in the blood stream) and other technical artifacts (e.g. error rates of NGS) that restrict the analytical sensitivity of tumour detection in plasma DNA.

In this study, we evaluate our targeted cfDNA sequencing approach that uses molecular barcodes – unique molecular identifiers (UMIs) for error correction. The developed assay spanning 77 genes (285.4 kb target region) is customized for BC, with focus on the commonly altered genes in BC as well as those with potential actionability^13,14^. To improve variant calling in ctDNA we developed a freely available bioinformatics pipeline (https://github.com/dkleftogi/cfDNA_AnalysisPipeline) that enables sensitive detection of SNVs and small insertions/deletions, accurate identification of copy number variations (CNVs) and evaluation of fragmentation profiles in cfDNA. We first assessed the performance of the method on reference samples. Then, in a proof of concept study, we applied the developed cfDNA assay and pipeline to detect genomic alterations in a cohort of 35 BC patients, and assessed the concordance of mutation calls with matched solid tumour sequencing.

## Materials and methods

### Patient recruitment and sample collection

Patients were recruited at National Cancer Centre Singapore in a prospective observational study approved by the Singhealth Centralised Institutional Review Board (2013/251/B and 2014/119/B) where blood specimens were collected from 35 patients with BC from 2014 to 2016. Signed informed consent was obtained from all patients. Matched primary and metastatic tumour specimens were also obtained for tumour sequencing, as well as buffy coat samples for matched normal sequencing. All methods were performed in accordance with relevant guidelines and regulations.

Retrospective review of medical and pathology records was performed to collect clinicopathologic details including patient demographics, tumour subtype via clinical testing, disease burden, and serum CA15-3 level where available. Patients with significant visceral disease burden requiring urgent chemotherapy+ /− targeted therapy rather than endocrine therapy were considered as having *high* disease burden. Patients with oligometastatic disease—in this series, with maximum of 5 sites of low volume disease, or stage 2 rather than 3 (moderate) for neoadjuvant cases, were considered as having *low* disease burden. Patients that fall in between the low and high categorization were considered to have *moderate* burden. The determination of estrogen receptor (ER), progesterone receptor (PR) and human epidermal growth factor receptor 2 (HER2) status by immunohistochemistry in this study was based on the latest recommendations at the time of the study by the American Society of Clinical Oncology and the College of American Pathologists^15,16^. ER and/or PR positive tumours that were HER2 negative were classified as hormone receptor positive (HR +)HER2-. Tumours with null expression in ER/PR and HER2 were classified as triple-negative breast cancer (TNBC) subtype. Tumours with positive HER2 expression (regardless of ER/PR status) were classified as the HER2-positive subtype.

Plasma samples from healthy individuals were also collected (study 2012/733/B). All individuals were considered healthy if they were not cancer patients at time of collection. Aliquots of 1–2 ml of plasma were used for this study.

### Sample preparation and sequencing

All plasma was separated from whole blood collected in EDTA tubes within 2 h of collection, and subsequently frozen at − 80 °C. Plasma DNA was extracted using the QiaAmp Circulating Nucleic Acids kit (Qiagen). FFPE sections were micro-dissected and DNA was extracted from these and frozen tissue using standard protocols. All DNA libraries were prepared using the Kapa Hyper Prep Kit (Kapa Biosystems, now Roche) using in-house designed library adapters with a random 8-mer proximal to the library index site (synthesized at IDT, Supplementary Note). Hybridization capture was done using an IDT Xgen Custom Panel of 77 genes (Supplementary Table 1) and reagents as per manufacturer’s instructions. Sequencing was performed on an Illumina Hiseq4000 (2 × 150 paired).

**Table 1:** Clinicopathologic characteristics of study cohort.

Characteristic	Number (n = 35)
Median age at study entry, in years	50 (36–75)
**Ethnicity**	
Chinese	23 (65.7%)
Malay	9 (25.7%)
Indian	1 (2.9%)
Others	2 (5.7%)
**Subtype**	
HR+/HER2-	18 (51.4%)
HER2+ (regardless of HR)	14 (40.0%)
TNBC	3 (8.6%)
**Stage**	
II	2 (5.7%)
III	3 (8.6%)
IV (all relapsed after prior non-metastatic diagnosis)	30 (85.7%)
**Number of prior systemic therapies (including non-metastatic)**	
0	5 (14.3%)
1 or more	30 (85.7%)
**Volume of Disease**	
Low	10 (28.6%)
Moderate	14 (40.0%)
High	11 (31.4%)
**Serum CA15-3 levels**	
Not raised	15 (42.9%)
Raised	18 (51.4%)
Not available	2 (5.7%)

Panel genes were chosen based on the literature, including the most commonly mutated genes from the TCGA, Sanger and METABRIC studies^17–19^ as well as the SAFIR-01 and MOSCATO trials^20^, with emphasis on genes that may have therapeutic implications.

### Processing of targeted sequencing cfDNA data with UMIs

**Figure 1:**
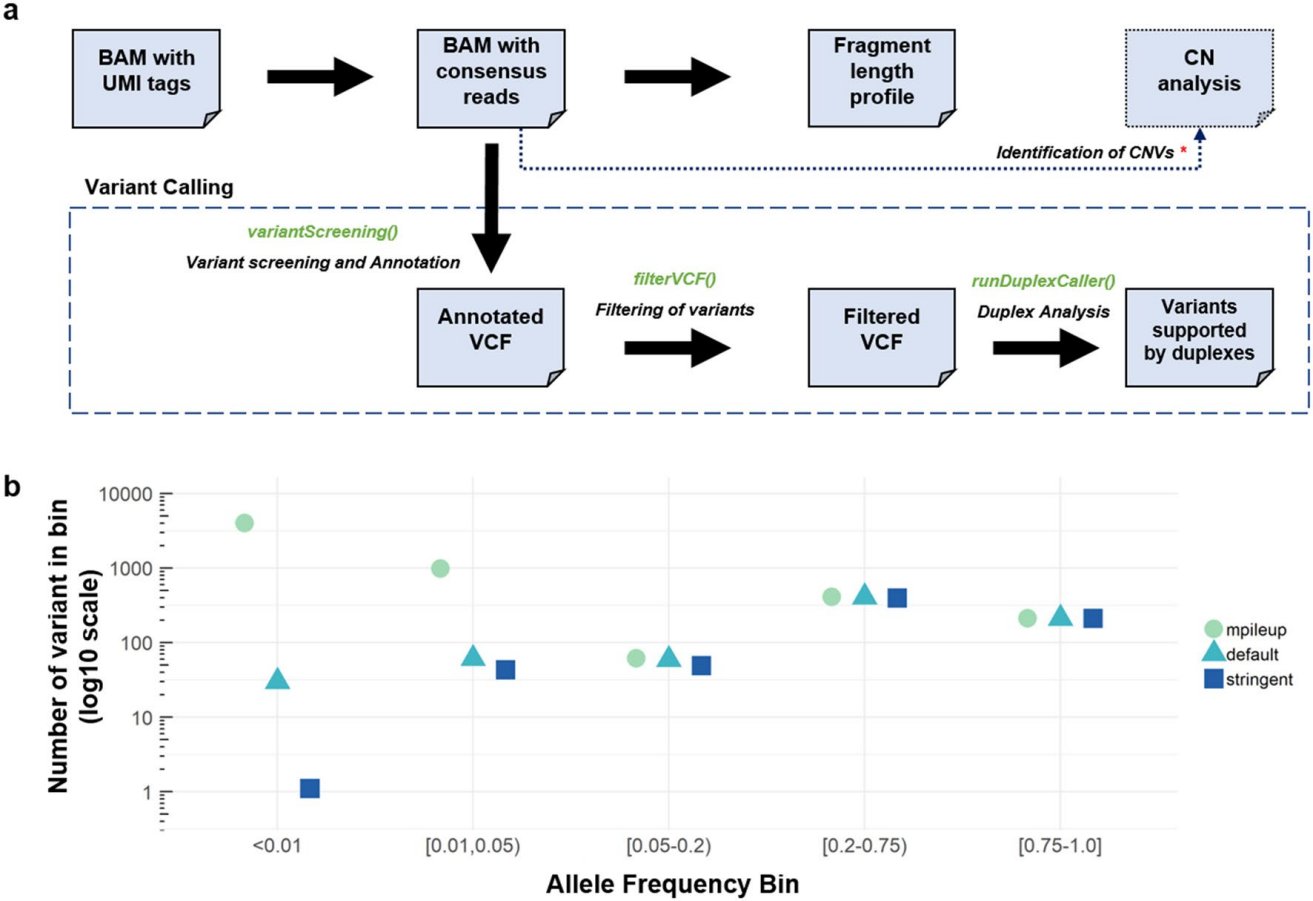
Outline of pipeline development. (**a**) Flowchart of developed pipeline, which includes Python functions() for each step available in our repository. CNV calling is available separately. (**b**) Number of variants called in healthy plasma samples (n = 20) in different frequency bins using a baseline approach (mpileup), default settings on our pipeline and stringent settings.

FASTQ files from sequencing of plasma samples were pre-processed to incorporate unique molecular identifiers (UMIs) into the fastq header, and then aligned to the hg38 reference genome using bwa-mem^21^ (version 0.7.15). Data from different lanes was merged to a single BAM file using GATK (version 4.1.1). The developed pipeline (Fig. 1a, function process Sample) was used for UMI-aware deduplication based on the fgbio package (version 0.8.1). Reads with the same UMI were grouped together allowing one base mismatch between UMIs with minimum mapping quality 20. Consensus sequences were generated using the “adjacency” function by discarding groups of reads with single members^22^. Quality statistics and on-target analysis of coverage (Supplementary Table 2) were obtained using samtools^23^ (version 1.3.1) and bedtools^24^ (version 2.18).

**Table 2:** Mutations found in known cancer-related genes by cfDNA sequencing.

Patient	Status	Anatom. stage	TB	CA 15-3	APC	ATM	BRCA1	BRCA2	EGFR	ERBB2	JAK2	KMT2C	MAGI3	MAP3K1	NF1	PIK3CA	PIK3R1	RET	TP53	Other
BC050	N	II	L	NA						R814H (0.007)									R273C (0.012)	
BC083	N	II	L	NA																
BC017	N	III	M	N																AKT1 S122L (0.006) AKT1 E151Q (0.006)
BC002	N	III	M	H													D643G (0.007)	Q44* (0.005)		MET A1239V (0.006) DOT1L G1014R (0.011)
BC003	N	III	M	H			L440* (0.011)									H1047R (0.57)			I195F (0.46)	
BC058	M	IV	L	N																
NTT00018	M	IV	L	N																
BC033	M	IV	L	N															S241C (0.006) L194X (0.003)	
BC042	M	IV	L	N															C238G (0.034)	AURKA S53Y (0.016) AURKA Q34K (0.018) AURKA Q74* (0.019) AURKA Q29K (0.02) AURKA S123* (0.035)
BC116	M	IV	L	N															S106R (0.047)	ERBB3 V104L (0.033)
BC095	M	IV	L	N			R1856Q (0.011)										D68N (0.250)			JAK1 P861Tfs (0.005) FGFR1 D166del (0.013) TSC2 S1764N (0.013)
BC062	M	IV	L	H																
BC072	M	IV	L	H						L755S (0.023)		S3585* (0.061)	S218C (0.04)			447-455del (0.045)			E286G (0.097)	KRAS A59T (0.018) GNAS P459R (0.397)
BC014	M	IV	M	N																
BC026	M	IV	M	N					T785N (0.008)										Y220C (0.016)	
BC031	M	IV	M	N														D58N (0.006)		
BC046	M	IV	M	N											D2077A (0.005)					
BC056	M	IV	M	N															R209X (0.005)	
BC098	M	IV	M	N	E129Q (0.016)			A2951T (0.024)										Q681E (0.022)		
BC013	M	IV	M	H																ARID1A A615fs (0.003)
BC028	M	IV	M	H			Y856H (0.005)							P403Tfs (0.007)						
BC054	M	IV	M	H					R680Q (0.007)											GATA3 S405Rfs (0.065)
BC100	M	IV	M	H											G772E (0.061) N773K (0.061)				E286K (0.33)	SMO R763* (0.005)
SB00005	M	IV	M	H												H1047R (0.035)				
BC021	M	IV	H	N																FBXW7 G477S (0.205)
BC040	M	IV	H	N	R1676T (0.016)					G727A (0.31)		D2297N (0.007)	Y1041H (0.24)			I112N (0.34)			Q331* (0.009) Q192* (0.49)	SMAD4 R135* (0.012) CDH1 175-splice (0.426)
BC068	M	IV	H	H																GATA3 P409Ffs (0.217)
BC089	M	IV	H	H		E609D (0.045)					S280* (0.006)					H1047L (0.124)			R282W (0.137) H214P (0.005)	MPL R229T (0.008) ATR R2337T (0.013) MPL E336Q (0.06)
BC092	M	IV	H	H				K3326* (0.008)			I724T (0.014)			M312L (0.014)						ABL1 G725S (0.017)
BC094	M	IV	H	H											R416* (0.257) E1276* (0.179)					ALK R551Q (0.006) KDR S1021L (0.017) JAK1 E897K (0.02) TSC1 Q55* (0.275) TSC1 H189R (0.281)
BC102	M	IV	H	H		I2914V (0.008)	M1673I (0.011)		R574W (0.008)										L111Ffs (0.65)	FLT3 D324N (0.005)
BC114	M	IV	H	H															R284Q (0.32)	
BC117	M	IV	H	H							R588X (0.124)					V344G (0.027) E542K (0.022)			R213* (0.41)	NOTCH1 G2535D (0.256)
CYK00017	M	IV	H	H															R248W (0.13)	
GJ00025	M	IV	H	H		E632Q (0.17)										C420R (0.53)			L194R (0.37) S241Y (0.75)	SMO L426V (0.195)

Variants from the plasma of 35 BC patients in recurrent genes are reported here. VAFs are in parentheses. Patient status refers to neoadjuvant (N) or metastatic cases (M). Tumour Burden (TB) has levels (L)ow, (M)edium and (H)igh and CA15-3 has levels (N)ormal and (H)igh or not available (NA). Patients are sorted by anatomical stage, tumour burden and CA15-3 levels.

### Bioinformatics pipeline for sensitive and stringent detection of variants in cfDNA

To identify variants (SNVs and small insertions/deletions) in cfDNA, we developed a two-step bioinformatics pipeline based on freely-licensed bioinformatics software. In the first step (Fig. 1a, functions variantScreening, filterVCF), the developed pipeline applies variant screening using VarDict^25^. In the second step (functions runDuplexCaller), VarDict’s filtered vcf file is used for further filtering using duplexCaller^12^. duplexCaller identifies variants supported by pairs of paired-end reads (i.e. families with different UMIs) that are mapped to the same genomic coordinates but with complementary sequencing orientation (i.e. one family in the forward strand and the other family in reverse strand). Variants supported by this configuration (denoted as “duplex pair reads” in duplexCaller’s original publication^12^) are more likely real predictions, which increases stringency and enables massive reduction of false positive variant predictions^12^. For clarity, we will refer to these as having “double-strand support”, because the adapter design does not give direct evidence that both read families originate from the same molecule.

### Determination of pipeline parameters

To assess sensitivity and specificity of the developed variant calling pipeline, we evaluated variant calling on commercial reference samples with specified variants and known allele frequencies (Seracare Seraseq ctDNA mutation mix—0.5%, 1% and WT). Thirty-seven variant positions (25 SNVs, seven insertions and five deletions) were covered in our panel. Samples were prepared as above, on a larger hybridization panel (226-genes), but with sequencing analysis restricted to the 77-gene panel set. We used the 0.5% and 1% VAF samples to assess sensitivity and the WT samples to assess specificity. We note, however, that the product specifications report that variants were detected by ddPCR in the WT sample (range 0–0.1%). Variants were called using ‘default’ parameters: filtering criteria of base quality 30, minimum coverage 100, at least three reads supporting alternative alleles, minimum signal to noise ratio 4 and mean position of variant in read greater than 10.

We also used sequencing of plasma samples from healthy individuals to evaluate the utility of our pipeline steps to remove false positives, as well as to fine-tune the parameter selection to increase specificity. Plasma samples from 20 individuals were sequenced and processed as above. Variants from these samples were called from UMI-consensus reads using a baseline approach (samtools mpileup to count alternate alleles), as well as under default parameters (as above) and under ‘stringent’ parameters, where minimum signal to noise ratio 20, and mean position of variant in read greater than 15.

### Variant calling in patient cfDNA

For BC patients, we used the abovementioned ‘stringent’ parameters to call variants, with these additional filters: (a) removal of all variant predictions also found in the plasma of two or more healthy individuals, in order to further eliminate alignment artifacts^26^, (b) selection of variants with damaging impact (MODERATE or HIGH) as annotated by Ensembl VEP^27^ and (c) removal of all variant predictions which are also found in matched buffy coat sequencing. Buffy coat sequencing data for each patient was aligned and duplicates removed as part of tumour sequencing (below), then alternate allele read-counts tallied using samtools mpileup. Patient plasma variant predictions were removed if there were more than zero alternate allele reads or less than 100X coverage in buffy coat sequencing.

### Processing and variant calling of sequencing data from solid tumour

Targeted sequencing data without UMIs from solid tumour samples were aligned to hg38 reference genome using bwa-mem (version 0.7.15). Data from different lanes were merged using GATK version 4.1.1 and duplicates were removed using GATK MarkDuplicates function. Quality statistics and on-target analysis of coverage (Supplementary Table 2) was performed using samtools (version 1.3.1) and bedtools (version 2.18).

Variants were called using a pipeline^28^ that uses Mutect2 variant caller^29^ with Platypus^30^. Mutect2 was first run with default parameters on all primary, metastatic (when available) and normal samples of every patient. Then, we used the VCF files returned by Mutect2 as priors to Platypus with zero posterior probability, and jointly called variants. To identify somatic variants in solid tumour samples for concordance, we required (a) minimum coverage of 50 reads, (b) at least 3 reads supporting the variant, (c) zero supporting reads in the matched normal sample with minimum coverage of 100 reads in the matched normal. To identify variants from tumour only, we also required that the quality filtering flag returned by Platypus either be ‘PASS’, ‘Q20’, ‘QD’, ‘alleleBias’ or ‘HapScore’.

### Identification of copy number variations (CNVs)

CNVs were called in ctDNA and solid tumour samples using CONTRA with the Null Distribution Estimation (NDE) workflow^31^ using a multimodal distribution instead of the unimodal distribution used by default. We took the best fit between a bimodal and trimodal distribution as determined by the Akaike information criterion (AIC) estimator and fed the model parameters into the software’s threshold cutoffs for CNV identification. All other parameters of CONTRA remained unchanged.

### Estimation of cfDNA fragmentation profiles

We also developed a function to characterize fragment length profiles of cfDNA samples (function fragmentLenAnalysis). The pipeline takes as input BAM files deduplicated with UMIs and uses pysam libraries (https://pysam.readthedocs.io/en/latest/index.html) to extract the fragment length values based on the TLEN sam flag of all read pairs mapped to the target region. We considered read pairs with minimum mapping quality 10, and excluded read pairs where mates were mapped to different chromosomes. The observed data were binned and normalized by the total number of read pairs sequenced in the sample. Following this procedure, we generated density profiles for all BC patients and healthy individuals. All reads from all healthy individuals were pooled to generate a combined fragment length profile that was used as a reference.

### Code availability

The bioinformatics workflow described in this study can be downloaded from our GitHub repository (https://github.com/dkleftogi/cfDNA_AnalysisPipeline). An overview of all functions is in Fig. 1a. We provide a collection of scripts written in Python for UMI-aware BAM file deduplication, mutation calling and fragment length analysis as well as a Conda virtual environment to resolve dependencies with existing packages.

## Results

### Patient and cohort characteristics

Of the 35 patients included in the study, 30 cases were metastatic with plasma samples taken prior to commencement of a new line of palliative systemic therapy (all subtypes). For 28 of these, both matched primary and metastatic specimens were sequenced. The remaining two did not have available specimens (BC058 metastatic and BC0098 primary). Plasma and primary specimens from five patients (three at stage III, and two at stage II) about to commence neoadjuvant systemic therapy were also collected. For all patients, matched genomic DNA from buffy coat was sequenced. The demographic characteristics and clinical information for all patients included in the study are presented in Table 1 and sequencing coverage presented in Supplementary Table 2.

### Pipeline performance and parameter assessment

We developed a two-step pipeline to call variants from UMI-aware consensus-sequencing reads. In the first step, variants are called using VarDict and filtered, and in the second step, variants are evaluated for having double-strand support using duplexCaller.

Using reference samples with known variant allele frequency (VAF), we evaluated the performance of the pipeline at both steps. At the first step, we observe that all 37 variants are called in both the 0.5% and 1% VAF samples (100% sensitivity), but also 9 variants in the WT sample (i.e. false positives, 76% specificity). By including duplexCaller in the second step, we see that all false positives are eliminated (100% specificity), but that sensitivity drops to 76% (28/37 variants) at 1% VAF, and 62% (23/37 variants) at 0.5% VAF. Next, using the same reference and WT samples we compared our performance with three state-of-the-art variant calling methods namely SiNVICT^32^, deepSNV^33^, and MutScan^34^. SiNVICT detected 5/37 mutations (13.5% sensitivity) at the level of 0.5% VAF, and 13/37 mutations (35.1% sensitivity) at the level of 1% VAF, whereas it resulted to 1 false positive prediction (97.2% specificity). deepSNV detected 5/37 mutations (13.5% sensitivity) at a level of 0.5% VAF and 10/37 (27% sensitivity) at the level of 1%VAF, but we could not assess specificity in the WT since deepSNV requires as input paired tumour-normal bam files. Finally, MutScan that works directly on FASTQ files achieved high sensitivity of 94.5% (35/37 detected mutations) in both reference samples. However, when we assessed specificity in WT MutScan returned 15 FP calls which is translated to specificity of 59%. Taken together our comparison analysis indicate that the deployed variant calling pipeline achieves a very good trade-off between specificity and specificity compared to alternative publications, and it increases the confidence that the identified variants are true.

Then, since the number of positions in the reference sample was limited, we further evaluated our variant calling on plasma from a set of healthy individuals (n = 20), with the assumption that these samples should not have significant low-frequency variants. We compared our pipeline results (‘default’ settings) against results using a minimal allele-counting approach (samtools mpileup) and found that at VAF > 5%, the number of variants called were very similar (total of 678 and 682 respectively, Fig. 1b). Variants in this frequency range are most likely to be true. However, we would expect few to no variants below 5%, barring clonal haematopoiesis events, and any called in this range are most likely to be false. We observe that at VAF < 1%, the baseline approach identifies 3971 potential variants, but our ‘default’ pipeline finds only 30, which is an error reduction of approximately 132 times. We tuned the pipeline filtering parameters for further error reduction and found that increasing the required signal to noise ratio, as well as requiring the variant to be found further from the edges in sequencing reads (Methods) allowed us to increase our stringency such that only 1 variant is called at VAF < 1%. With these parameters, we also see that sensitivity is mostly maintained in the reference sample, with only one fewer variant detected at both VAFs, to give a sensitivity of 73% at 1% VAF and 59.3% at 0.5% VAF. Variant calling on BC patient plasma was thus done using these ‘stringent’ settings.

### Variants detected in cfDNA samples

Our NGS assay combined with the proposed bioinformatics pipeline for variant calling provides sufficient sensitivity to detect tumour-associated variants in 30 out of 35 samples with median VAF of 0.02 (Table 2, Supplementary Table 3). Across 30 samples with detectable variants, a total of 98 variants were detected, with most of them (n = 63) already previously described in COSMIC. The variants detected using the deployed cfDNA assay highlights the capability of identifying biologically important mutations directly from cfDNA. Among the five patients with no detectable tumour variants in cfDNA, four of them had low tumour burden based on clinical and radiological information (Table 2).

As expected, known tumour suppressor or oncogenic genes such as *TP53* (found mutated in 16/35 patients), *PIK3CA* (7/35), *BRCA1* (4/35), *NF1* (3/35), *EGFR* (3/35), *ERBB2* (3/35), *ATM* (3/35) or *RET* (3/35) are found recurrently mutated in our cohort. Overall 18 out of 25 patients harbored more than two mutated genes.

### Concordance of cfDNA and sequencing of solid tumour sites

**Figure 2:**
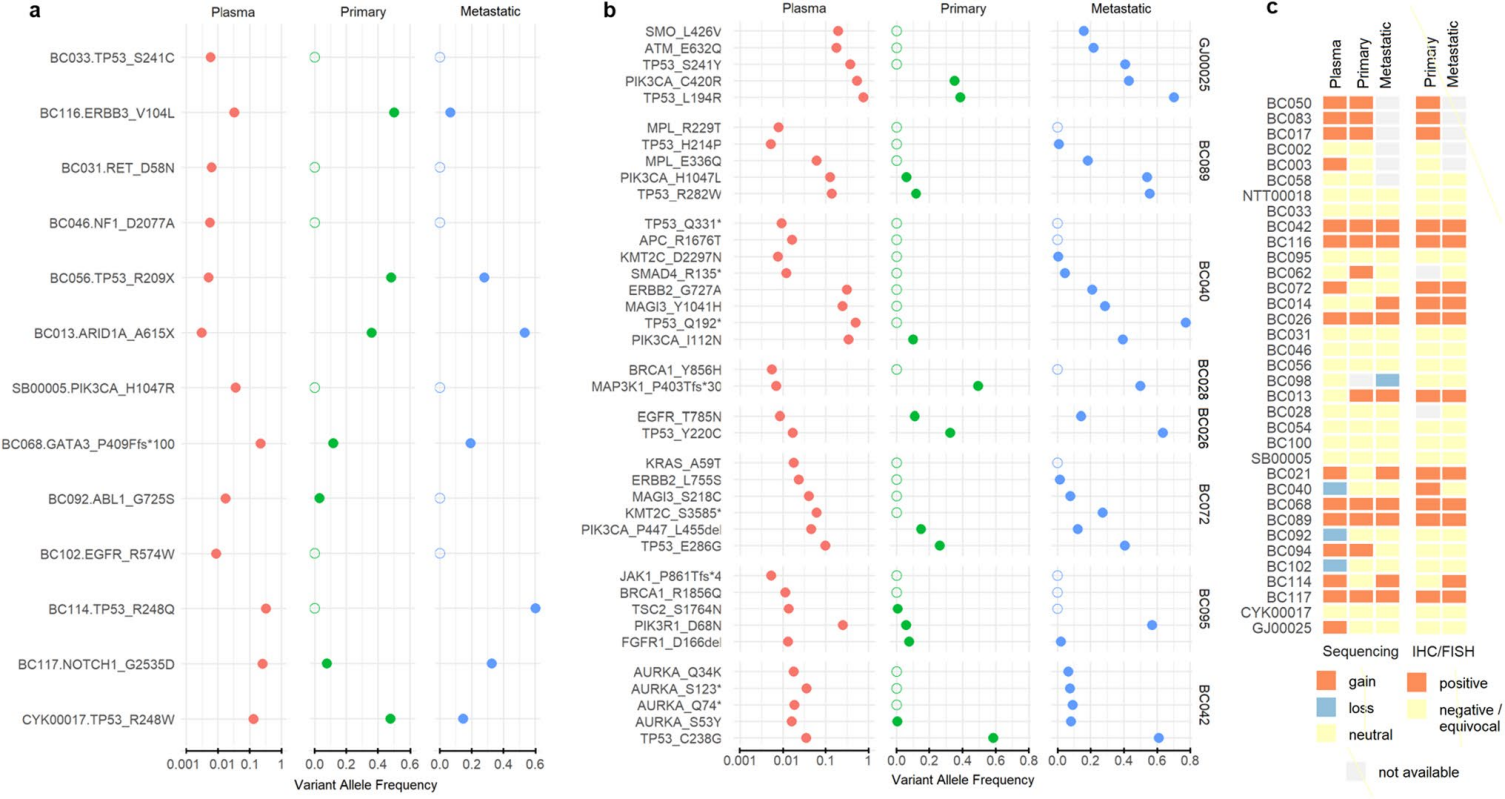
Concordance of variants identified by cfDNA and tumour sequencing. A set of 51 variants for which cfDNA, primary and metastatic tumour sequencing coverage was adequate was identified. VAFs of the variants in each sample type are shown, with plasma cfDNA variants shown on a log-scale. (**a**) Samples which only had a single variant in this set. (**b**) Samples with multiple variants in this set. Samples are ordered as in Table 2. (**c**) CONTRA results for CNV calling for plasma cfDNA, primary and metastatic tumour sites.

On the subset of 28 samples which had both primary and metastatic tumour available, we assessed the concordance and discordance of mutations detected in cfDNA with that in solid tumour sites. Our cfDNA assay detected 84 mutations across these plasma samples (Supplementary Table 3). Due to challenges in FFPE sequencing and resultant lack of sufficient coverage, 33 positions could not be evaluated in the solid tumour. We thus restricted this concordance analysis to the 51 mutations that had coverage of more than 50 reads in both the primary and metastatic samples. Using this set of 51 callable positions we identified 20 mutations (~ 39.2%) that were concordant between cfDNA, primary and metastatic samples, and 17 mutations (~ 33.3% of all cases) that were concordant between cfDNA and metastatic sites but not found in primary sites, which is consistent with the patient clinical progression at time of sampling and could represent emergent or subclonal mutations. Figure 2 shows the VAFs of variants found in both the plasma and tissue sequencing, and we see that most variants found in plasma sequencing are well represented in the tissue (VAF > 10%), but a number are found at subclonal levels, with four found at tissue VAF < 1%. Another two mutations were concordant between plasma and primary sites but not found in the metastatic tumour.

We also detected 12 mutations (~ 23% of all variants) in plasma with median VAF of ~ 0.008 (range 0.005–0.03) that were not present in either primary or metastatic sites. Many discordant mutations found are in important oncogenes such as APC (R1676T), JAK1 (860–861,-/X), PIK3CA (H1047R) , KRAS (A59T), BRCA1 (1856Q, Y856H), EGFR (R574W), MPL (R229T) ,NF1 (D2077A), RET (D58N) and TP53 (S241C, Q331*) highlighting the effectiveness of the developed cfDNA assay to monitor potential emergent cancer alterations directly from cfDNA, even when mutation-bearing molecules are very rare in plasma. Together, our data show that cfDNA screening using the developed assay addresses the majority of variants found by solid tumour sequencing (overall ~ 77% concordance with at least one solid tumour site), including in BC patients with low tumour burden. However, the results of this analysis should be interpreted with caution, because while emergence of new variants in metastatic tumours is not unexpected, we cannot completely rule out that the absence of variants in primary sites is due to technical reasons like sampling or inferior sample quality of FFPE-extracted DNA from solid tumour sites.

Finally, we assessed variants called in solid tumour sites and not in cfDNA, for which there were only seven variants. Two (variants in genes *PIK3CA* and *TP53*) were from the tumours of patients with non-metastatic disease, two (variants in genes *MAGI3* and *EZH2*) were from primary tumour only, and three (variants in genes *APC*, *TP53* and *MDM4*) were from metastatic tumours only. Five out of seven variants not detected in cfDNA had zero support of the alternative alleles in cfDNA, except for two that had sufficient supporting reads (13 and 29 reads for variants TP53 Y236R and MDM4 A42V respectively), but no double-strand support. Such cases are likely false negatives of our highly-stringent variant calling pipeline.

### *ERBB2* CNV analysis and HER2 subtype classification in BC

We investigated whether we could use the developed cfDNA assay to detect CNVs in BC. We applied a customized version of the CONTRA algorithm to infer CNV profiles using the reads generated by targeted hybrid capture cfDNA sequencing. We focused on the *ERBB2* (HER2) oncogene to identify detectable amplifications that may be used for HER2 subtype classification. For validation purposes, all patients in the cohort had previously undergone HER2 testing mainly via immunohistochemistry, with fluorescent in-situ hybridization (FISH) testing performed for equivocal immunohistochemical results (Supplementary Table 4). Fourteen of them were found to be HER2+, whereas 18 patients were HR+HER2− and 3 were Triple-Negative (TNBC). Our cfDNA approach detected correctly 12 of HER2+ patients (*ERBB2* amplified) and 18 of HER2−/TNBC patients, whereas it misclassified 5 patients. From the misclassified cases, 3 patients were falsely predicted to be HER2+, and 2 patients were falsely predicted to be HER2-, classification performance that could be translated to 80% positive predictive value (Precision), 85.7% true positive rate (Sensitivity) and 85.7% true negative rate (Specificity). Using the same algorithm, we also generated *ERBB2* CNV profiles using sequencing data from primary and metastatic solid tumour sites (Fig. 2c). We note that the *ERBB2* amplification was also detected correctly in 10 out of 14 patients using data from matched metastatic tumour sites, and in 7 out of 14 patients using data from primary tumour sites. In total, our findings using orthogonal sequencing and immunohistochemistry validate further the ability of our cfDNA assay to identify *ERBB2* CNVs in BC without prior knowledge of tumour sequencing.

### cfDNA fragmentation profiles of BC patients

**Figure 3:**
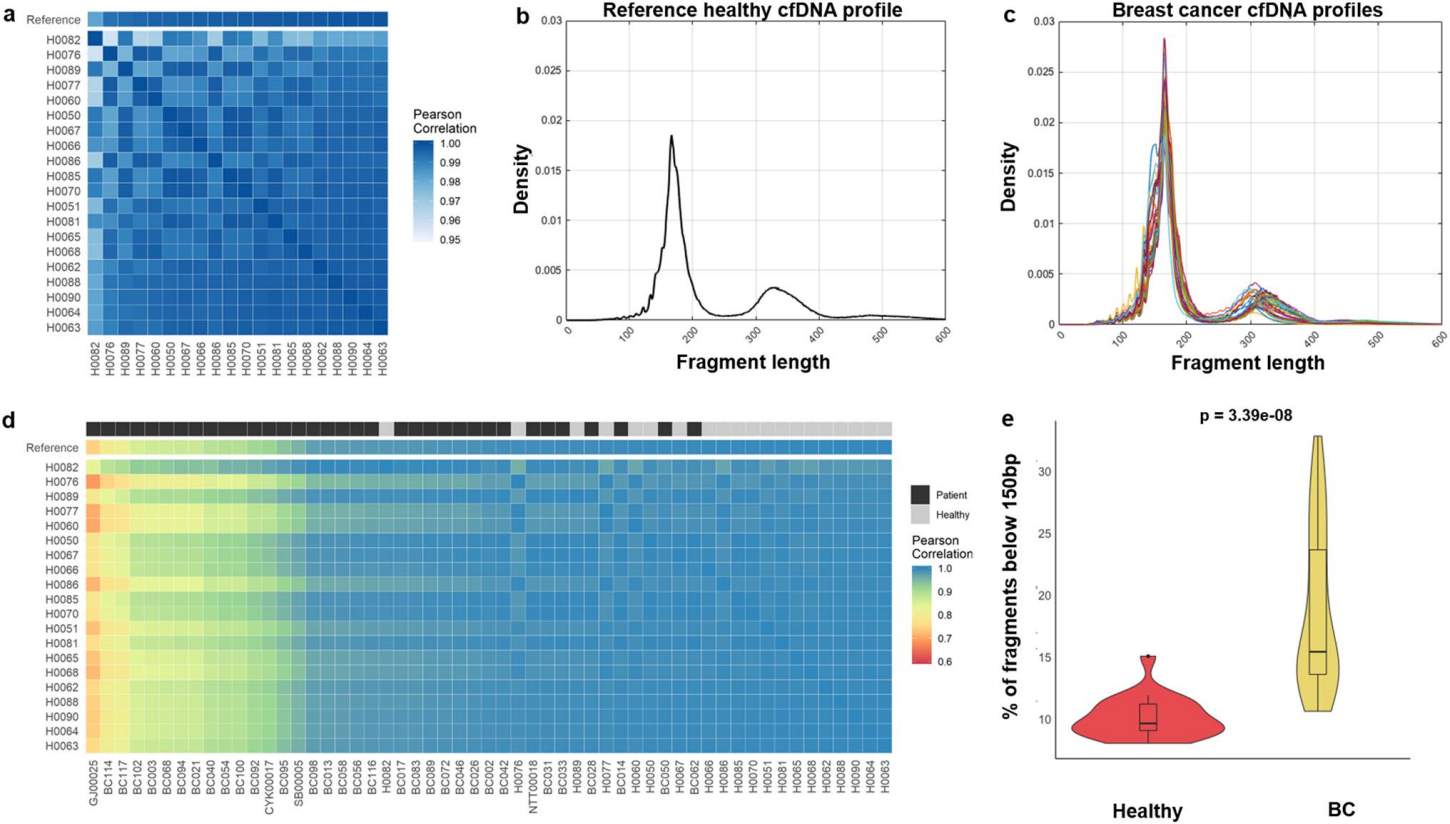
Analysis of cfDNA fragmentation profiles in BC. (**a**) Pairwise correlation of fragmentation profiles between 20 healthy individuals. All profiles are almost identical with minimum Pearson’s r > 0.95. (**b**) The reference cfDNA fragmentation profile complied using data from 20 healthy individuals. (**c**) cfDNA fragmentation profiles of 35 BC patients. (**d**) Pairwise correlation between 20 healthy individuals (y-axis) vs. 35 profiles of BC patients and 20 profiles of healthy individuals. Profiles arranged in ascending correlation with the reference profile. (**e**) The proportion of short cfDNA fragments (below 150 bp) detected in 20 healthy individuals is much lower compared to the proportion of short fragments detected in 35 BC patients. The distributions were compared using the Wilcoxon rank-sum test and *p* values are shown.

Recent analyses of the fragment sizes of cfDNA of patients with cancer suggest altered fragmentation profiles compared to healthy individuals^35^. Here, we used our developed cfDNA assay to characterize the fragment length profiles of 35 patients with BC and to explore its potential as a biomarker for disease monitoring. First, we analysed the cohort of 20 healthy individuals and found near-perfect correlation (> 0.95 Pearson’s correlation coefficient) between different healthy profiles (Fig. 3a), which indicates that cfDNA fragmentation profiles can be quite consistent across different healthy individuals. Based on this observation, we summarized the healthy profiles by combining all reads and estimating an average reference profile (Fig. 3b). In comparison, the BC patient profiles varied across patients, with some showing a characteristic shift of fragment length to be shorter (Fig. 3c). We then calculated the pairwise correlation between each of the healthy and patient profiles. The healthy profiles maintain a high correlation between each other, whereas the cfDNA profiles of BC patients were distinctly different (Fig. 3d). We also observe that the proportion of fragments below 150 bp is much higher in BC patients (Fig. 3e, Wilcoxon rank-sum test *p* = 3.390e−08) compared to the healthy samples, which is concordant with the results from other studies which used mainly low-pass WGS samples from treatment naïve or early stage patients^35,36^. To further confirm this observation, we compared the distribution of fragment lengths in variant-bearing reads from patient samples, and found it was distinctly different from the healthy reference (Supplementary Fig. 1, r = 0.75 and *p* = 1.383e−117).

### Correlation of cfDNA characteristics with clinical characteristics

**Figure 4:**
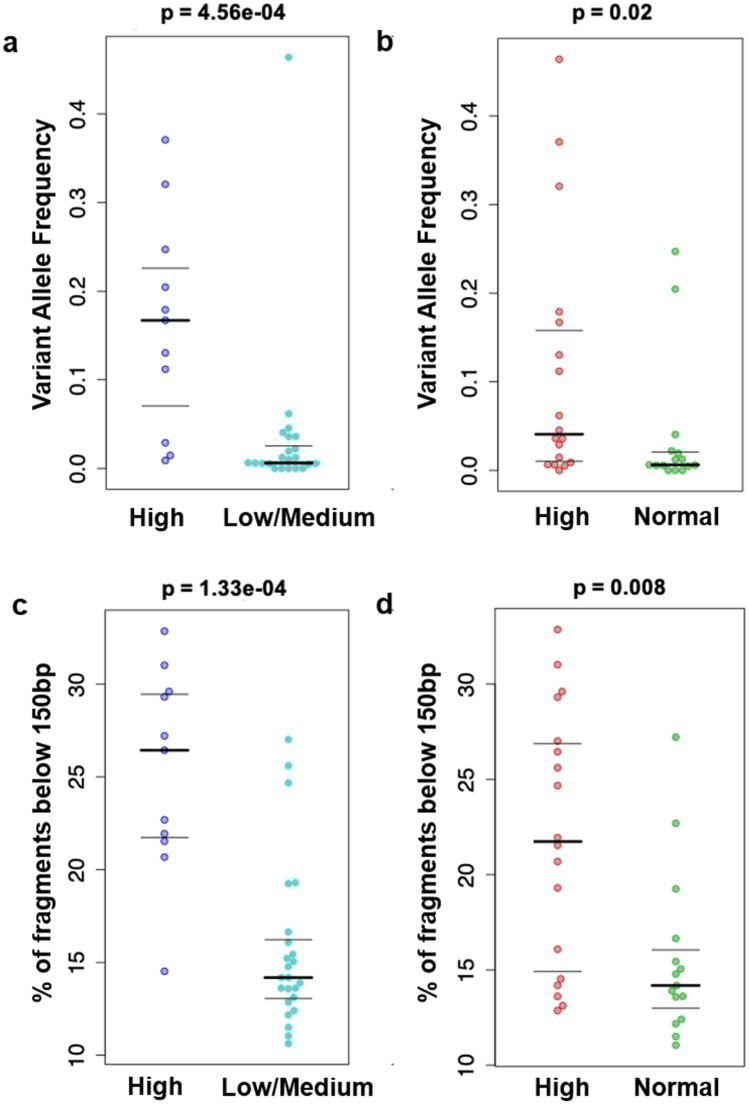
Association between genomic alterations detected in cfDNA and clinical information. Comparison of median VAF in patients with (**a**) high and low/medium tumour burden and (**b**) high and normal CA15.3 and comparison of the proportion of cfDNA fragments below 150 bp in the same (c,d). Comparison was done with the Wilcoxon rank-sum test and *p* values are shown.

To investigate the translational potential of the identified variants in cfDNA we performed correlation analysis using the cancer antigen 15-3 (CA15-3) tumour marker, which is frequently used in routine clinical practice. We computed the median VAF of all patients in the cohort and stratified them based on their tumour burden from clinical and imaging reports. We found that patients with high volume of disease (such as patients with widespread metastatic disease or in visceral crisis), harbor variants in cfDNA at much higher VAF levels compared to tumours of low (clinical stage or oligometastatic disease) and medium burden (burden of disease intermediate between high and low disease burden) (Fig. 4a, Wilcoxon rank sum test *p* = 4.56e−04). We also found that patients with high levels of the CA15-3 harbor mutations in cfDNA at higher VAF levels compared to tumours of normal CA15-3 levels (Fig. 4b, Wilcoxon rank sum test *p* = 0.02).

Our fragmentation analysis also indicates that fragmentation profiles of cfDNA in BC integrate genomic and epigenomic (i.e. nucleosome positioning) features that could serve as novel biomarkers in clinical settings. To investigate this hypothesis in our cohort, we associated our findings with CA15-3 levels and tumour burden, which was assessed as above. We found that patients with high tumour burden have a much higher proportion of shorter fragments compared to patients with low or medium tumour burden (Fig. 4c, Wilcoxon ranksum test *p* = 1.33e−04). We also found that shorter fragments are also proportionally higher in patients with elevated CA15-3 levels, compared to patients with normal CA15-3 levels (Fig. 4d, Wilcoxon ranksum test *p* = 0.0088). Consequently, our findings based on fragmentation profiles are in concordance with the results obtained from routine cancer biomarkers. We note that this is similar to the results obtained with median VAF, and indeed, we observe that the deviation of the patient fragment length profile from the reference (1—correlation between patient profile and reference profile) is highly correlated with median VAF (Pearson’s r = 0.793, *p* = 1.477e−08, Supplementary Fig. 1).

## Discussion

Several recent studies have demonstrated the ability of cfDNA sequencing to provide early prognostication, better molecular profiling and monitoring of disease dynamics. We developed a new error-corrected cfDNA sequencing approach with a panel customized for BC, in combination with publicly available bioinformatics strategies for the identification of tumour-associated genomic alterations. The developed NGS assay covers 77 known cancer-related genes (285.4 kb target region), providing the opportunity to elucidate genomic alterations with significant clinical value without prior knowledge of tissue sequencing.

Usage of UMIs for error correction, combined with variant calling based on variants with double-strand support, allowed us to detect low VAF variants in ctDNA samples with reduced number of false positives. We detected variants in 30 out of 35 cfDNA samples with ~ 64% of variants already reported in COSMIC. The majority of detected mutations in cfDNA were concordant with sequencing of solid tumour sites, and more potential mutations were also identified, many of them at VAF below 1%. The presence of these variants are consistent with tumour evolution hypotheses, where multiple low-VAF clonal and sub-clonal alterations may be the basis of resistance to several lines of chemotherapy. Although our study is limited in orthogonal validation (e.g. ddPCR), we have confidence in our calls because of the stringent parameters used in our pipeline.

We also observe an advantage of using ctDNA in cases where archival FFPE samples were not suitable for sequencing. In our cohort, nearly 30% of called plasma variants could not be evaluated for concordance in older primary tissue. Here it is a study limitation, but also an indication of the potential fraction of archival FFPE samples that may be unsuitable for molecular testing.

Recent studies have highlighted that mutations in cfDNA may be due to somatic mutations in clonal haematopoiesis (CH) and not ctDNA. In our study, we have mitigated this by removing any calls also found in matched buffy coat. We note, however, that our buffy coat sequencing is limited (range of average coverage: 124–483X). In the 35 variants for which we find no matched variant in the tumour (whether due to lack of sample or lack of coverage or lack of alternate allele), we find that in 24 of them there is a 5% probability, based on the binomial distribution, that we have missed a potential CH variant due to insufficient coverage. It is important that future studies should have matched high depth sequencing, e.g. at > 918X coverage for 99% probability to sample a 0.5% variant, to more comprehensively characterize CH in samples.

Our assay was also effective on the detection of CNVs in important oncogenes such as *ERBB2* that are equally important for understanding tumorigenesis and deciphering tumour progression mechanisms. This opens opportunities for better tumour characterization, where sequential plasma samples can be collected to portray more accurately CNVs and variants across time.

Finally, our study reports on cfDNA fragmentation profiles in BC. Our data from 35 BC patients and 20 healthy individuals re-confirmed that cfDNA fragmentation profiles recapitulate genomic and epigenomic features that are in principle, different. These profiles also correlate well with the mutational load measured by VAF in cfDNA. Furthermore, we observed that the fragment length of mutated reads from cancer-associated variants have distinct differences from the fragment length of reads that derive from healthy individuals. The observed differences could be used to improve our understanding of tumour biology of circulating nucleic acids. They might be also used to deconvolute signals that might enable the development of tumour content estimation algorithms in plasma DNA. Importantly, our observations open new avenues for monitoring patients’ progression under treatment, and developing cancer biomarkers using cfDNA sequencing. Towards this direction, future investigations are required to establish whether the determination of cfDNA fragmentation profiles might provide prognostic value for patients with early stage BC.

However, similar to all other cfDNA sequencing studies, our approach has several limitations. First, ultra-sensitive detection of cancer especially at early stage patients is not always viable^3,5^. Low abundance of cfDNA in plasma combined with the inherent error rate of NGS might limit the applicability of the developed assay. Using UMI-libraries reduces errors from library preparation and PCR amplification but cannot remove it completely. Finding variants using duplexCaller likewise increases stringency, but is limited by counting statistics. As we observed in both the reference and patient samples, there are variants that are filtered out due to lack double-strand support are likely true positives, but for which the conditions (e.g. input amount, capture efficiency, sequencing depth) did not capture both input strands. This indicates there is room for future improvements, to fine-tune parameters to balance stringent and sensitive detection of variants, and we expect that our pipeline will greatly assist in finding this. In addition, the developed targeted assay is less powerful on the detection of structural variants (e.g. translocations or inversion) compared to whole exome or whole genome sequencing data. This is an inherent limitation of selective sequencing and further work may be done in the development of more accurate methods for CNV detection in targeted NGS data. Finally, the small cohort size and the absence of serial specimens for comparisons, limit our ability to associate the findings of this study with clinical outcome.

Overall, our results highlight the effectiveness of our pipeline, which echoes others in opening possibilities for longitudinal monitoring of cancer-genomic alterations directly from plasma, without the increased risk and cost of invasive needle biopsies. Importantly, our pipeline does not rely on statistical modelling for background noise estimation^37^, which require big cohorts of reference data (e.g. healthy) that are usually difficult to collect, and may not be easily extended or generalized (e.g. if the panel size increases). In contrast, our approach relies on the effective combination of existing bioinformatics tools (i.e., VarDict and duplexCaller) which is simple and fast. In addition, the pipeline’s parameters are adjustable by the user to fit the majority of possible applications. Considering that many alternative pipelines are not freely licensed but proprietary software, we believe that our pipeline presents a significant advancement that could serve as a paradigm for future research in ctDNA.

## Supplementary information

Supplementary file

Supplementary file

## Data Availability

The bioinformatics pipeline is freely available at https://github.com/dkleftogi/cfDNA_AnalysisPipeline. The NGS datasets analysed during the current study is available from the authors on reasonable request.
