# Supplementary material for: Detection of genomic alterations in breast cancer with circulating tumour DNA sequencing
Source: Sci Rep. 2020 Oct 8;10:16774. doi: 10.1038/s41598-020-72818-6 (PMC7544894; doi:10.1038/s41598-020-72818-6)
Supplement: Supplementary file 1 — Supplementary file [file 41598_2020_72818_MOESM1_ESM.docx]

**Detection of genomic alterations in breast cancer with circulating tumour DNA sequencing**

Dimitrios Kleftogiannis^1^, Danliang Ho^1,2^, Jun Xian Liew^1^, Polly Poon^1^, Anna Gan^1^, Raymond Chee-Hui Ng^2^, Benita Kiat-Tee Tan^3^, Kiang Hiong Tay^4^, Swee-Ho Lim^5^ , Gek San Tan^6^, Chih Chuan Shih^1^ , Tony Kiat-Hon Lim^6^ , Ann Siew-Gek Lee^7,8,9^, Iain Tan^1,2^, Yoon-Sim Yap^2,9,*^ and Sarah Ng^1,*^

^1^ Genome Institute of Singapore (GIS), Agency for Science, Technology and Research (ASTAR), Singapore, 138672, Singapore.

^2^ Division of Medical Oncology, National Cancer Centre Singapore (NCCS), Singapore, 169610, Singapore

^3^ Department of General Surgery, Singapore General Hospital (SGH), Singapore, 169608, Singapore

^4^ Vascular and Interventional Radiology Department, Singapore General Hospital (SGH), Singapore, 169608, Singapore

^5^ KK Breast Centre, Kandang Kerbau Women's and Children's Hospital, Singapore, 229899, Singapore

^6^Department of Anatomical Pathology and Translational Pathology Centre, Singapore General Hospital (SGH), Singapore, 169608, Singapore

^7^Division of Cellular and Molecular Research, Humphrey Oei Institute of Cancer Research, National Cancer Centre Singapore (NCCS), Singapore, 169610, Singapore

^8^Department of Physiology, Yong Loo Lin School of Medicine, National University of Singapore (NUS), Singapore, 117597, Singapore

^9^SingHealth Duke-NUS Oncology Academic Clinical Programme (ONCO ACP), Duke-NUS Medical School, Singapore, 169857, Singapore

* To whom correspondence should be addressed

**Supplementary Figure**


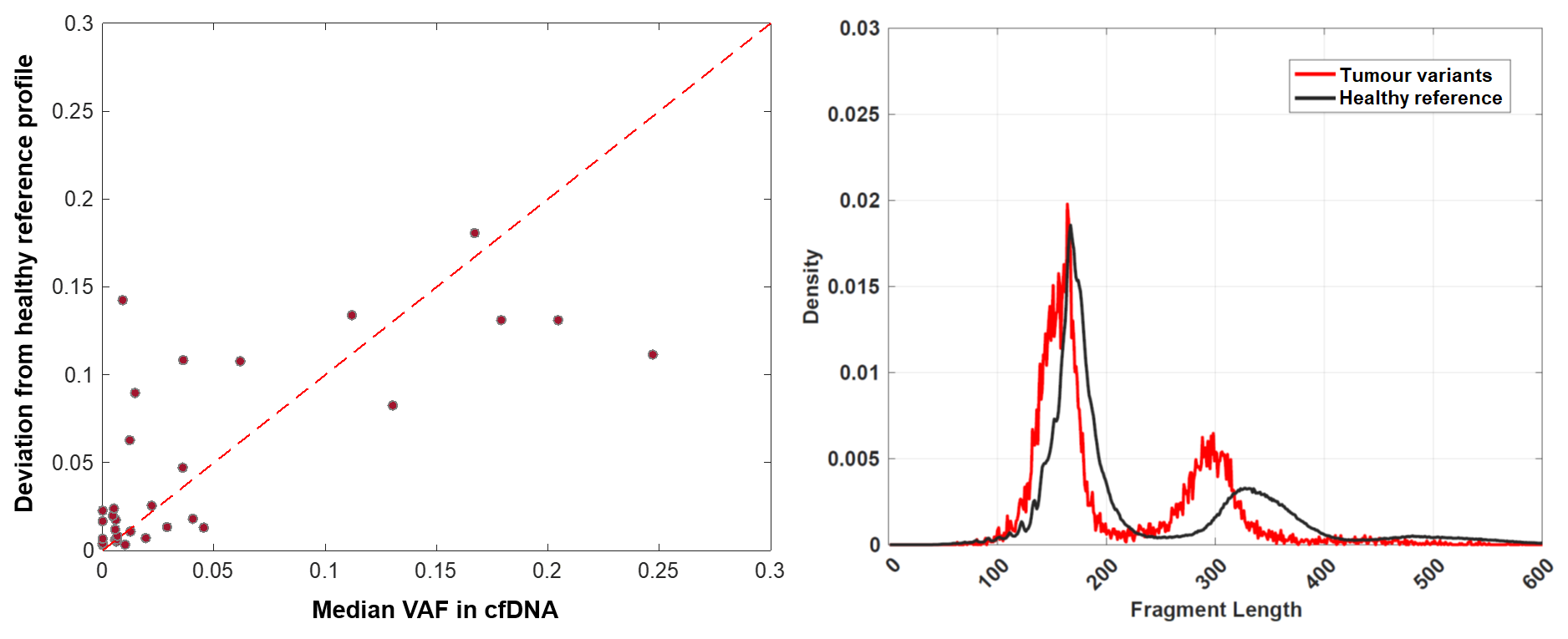


**Supplementary Figure 1. Analysis of fragment length profiles.** (Left) The deviation of BC profiles from the reference profile (1 – correlation between patient profile and the reference) is correlated with the median VAF observed in the same sample (Pearson’s correlation coefficient r=0.793 and p value= 1.477e-08). (Right) Distribution of the length of all mutation-bearing reads aggregated from all samples (red) against the healthy reference (black) (Pearson’s correlation coefficient r=0.75 and p value= 1.383e-117) .

**Supplementary Note**

**Adapter Design**


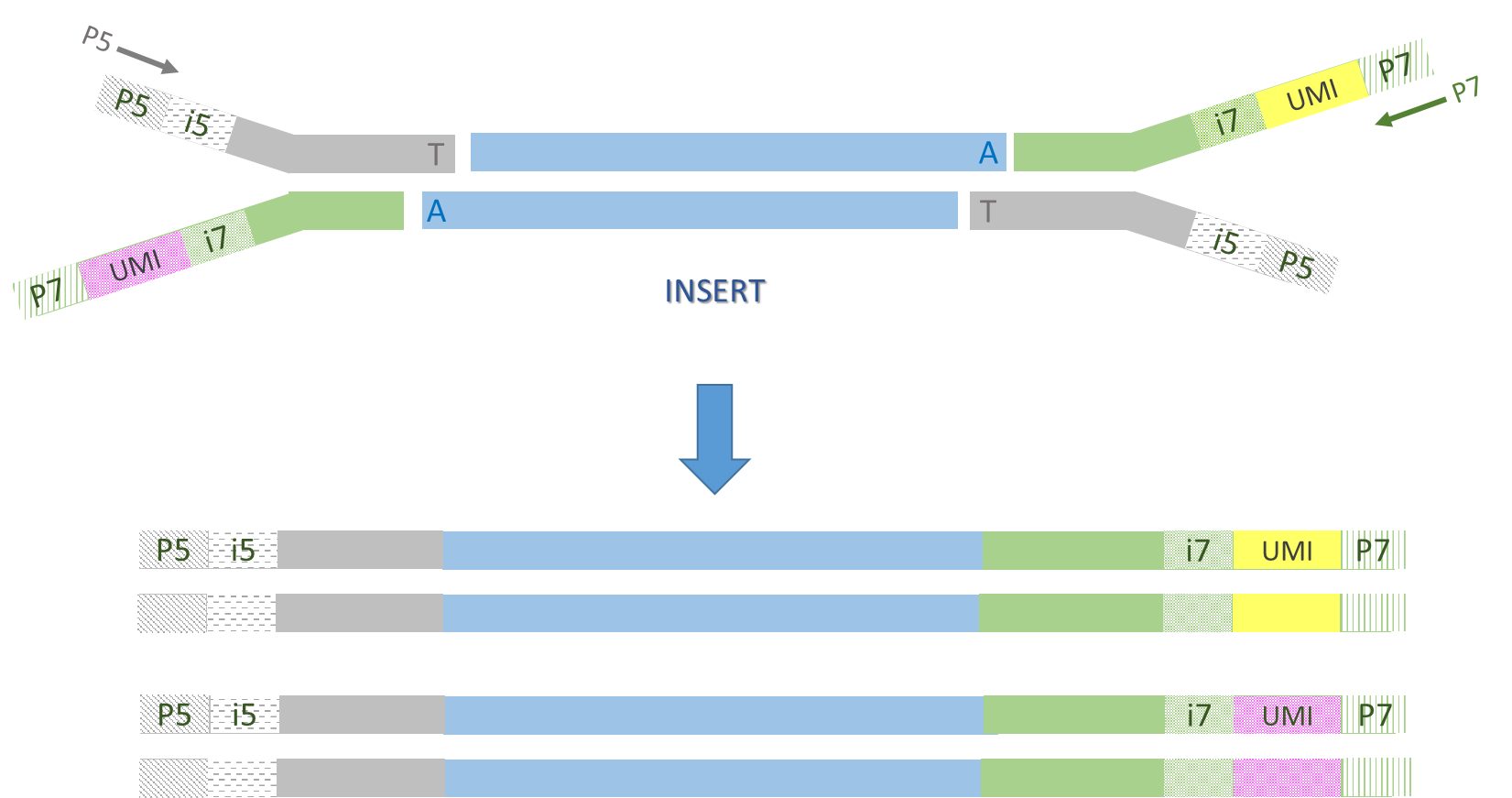


Our library adapters are based on the standard Illumina full-length Y-adapters, with the main difference that a unique molecular index (UMI) has been added downstream of the i7 index site. One strand (grey) of the adapter thus consists of the P5 attachment site, i5 (second index, optional) site and sequencing primer (R1) site including a T-overhang. The other strand (green) consists of the sequencing primer (R2) site, i7 (first index), single-stranded UMI and then the P7 attachment site. Both strands are synthesised separately and then annealed. The i5 and i7 indices used all are in-house designed to be edit distance 3 from each other and are not repeated. After ligation, the use of P5 and P7 primers in a PCR makes the library fully double-stranded, with each strand of each input molecule bearing a different UMI.

Apart from the library indices used, this design appears identical to the now commercially available IDT xGEN UDI-UMI adapters^1^. This scheme does not have the base-paired UMI that is necessary for direct molecular evidence of duplex tagging^2^.

**References**

1. xGen UDI-UMI Adapters | IDT. <https://sg.idtdna.com/pages/products/next-generation-sequencing/adapters/xgen-udi-umi-adapters>.
2. Schmitt, M. W. et al. Detection of ultra-rare mutations by next-generation sequencing. Proc. Natl. Acad. Sci. U.S.A. 109, 14508–14513 (2012)
